# Post-operative KEloids iRradiation (POKER): does the surgery/high-dose interventional radiotherapy association make a winning hand?

**DOI:** 10.1007/s11547-024-01756-4

**Published:** 2024-01-27

**Authors:** Jessica Franzetti, Stefano Durante, Federico Mastroleo, Stefania Volpe, Francesca De Lorenzi, Marco Rotondi, Chiara Lorubbio, Angelo Vitullo, Samuele Frassoni, Vincenzo Bagnardi, Raffaella Cambria, Federica Cattani, Andrea Vavassori, Barbara Alicja Jereczek-Fossa

**Affiliations:** 1 Division of Radiation Oncology, IEO European Institute of Oncology IRCCS, Via Ripamonti, 435, 20141 Milan, Italy; 2 Department of Oncology and Hemato-Oncology, University of Milan, 20122 Milan, Italy; 3 Department of Translational Medicine, University of Piemonte Orientale (UPO), 28100 Novara, Italy; 4 Department of Plastic and Reconstructive Surgery, European Institute of Oncology, IRCCS, Via Ripamonti, 435, 20141 Milan, Italy; 5 Department of Statistics and Quantitative Methods, University of Milan-Bicocca, Milan, Italy; 6 Unit of Medical Physics, European Institute of Oncology IRCCS, Milan, Italy

**Keywords:** Brachytherapy, Keloid, HDR-IRT, POIRT

## Abstract

**Purpose:**

To report the results involving post-operative interventional radiotherapy (POIRT) in a homogenous cohort of patients affected by keloid and treated at a single institution with the same fractionation schedule.

**Patients and Methods:**

Inclusion criteria were: surgery with a histopathological diagnosis of keloid, subsequent high-dose rate interventional radiotherapy (HDR-IRT)—12 Gy in 4 fractions (3 Gy/fr) twice a day—and follow-up period ≥ 24 months.

**Results:**

One-hundred and two patients and a total of 135 keloids were eligible for the analyses. Median follow-up was 64 [IQR: 25–103] months. Thirty-six (26.7%) recurrences were observed, 12-months and 36-months cumulative incidence of recurrence were 20.7% (95% CI 12.2–28.5) and 23.8% (95% CI 14.9–31.7) respectively. History of spontaneous keloids (HR = 7.00, 95% CI 2.79–17.6, *p* < 0.001), spontaneous cheloid as keloid cause (HR = 6.97, 95% CI 2.05–23.7, *p* = 0.002) and sternal (HR = 10.6, 95% CI 3.08–36.8, *p* < 0.001), ear (HR = 6.03, 95% CI 1.71–21.3, *p* = 0.005) or limb (HR = 18.8, 95% CI 5.14–68.7, *p* < 0.001) keloid sites were significantly associated to a higher risk of recurrence.

**Conclusions:**

The findings support the use of surgery and POIRT as an effective strategy for controlling keloid relapses. Further studies should focus on determining the optimal Biologically Effective Dose and on establishing a scoring system for patient selection.

## Introduction

Keloids are fibrotic lesions that results from an uncontrolled fibroblastic growth in the skin following surgeries, traumas, or minor stimulations [1]. However, in predisposed patients, keloids can develop spontaneously and, in some cases, a genetic susceptibility can also be recognized [2]. As an example, darker skin develops keloids fifteen times more likely than lighter one [3], and no keloids have been identified in albinos [4].

To date, the complex mechanism of keloid formation has not been fully elucidated and the pathological scar development may be due to either single nucleotide polymorphisms [5] or chromosomal changes [6]. Nevertheless, it is likely that many other genetic factors have not been recognized yet. Such as pathogenesis, even clinic is specific for keloids: scars present a tumor-like behavior and continuous growth, while patients can experience itch, pain and/or soreness. Of note, hypertrophic scars rarely extend beyond the original wound area and can even turn to normal skin within a few years.

Therapeutic options for keloids include topical treatments, such as cortisone injections or other medical procedures [7]; the local application of chemotherapy or physical treatment (e.g., cryotherapy or lasers) can also be a choice. Surgery alone can easily remove the keloid, but the risk of local recurrence is more than 50%, which emphasizes the need of adjuvant treatments, especially in case of larger lesions.

Since 1909, Radiotherapy (RT) with various techniques and energies was applied to treat keloids, such as photon- or electron-based External Beam RT (EBRT) [8]. Recently, several studies have supported the use interventional radiotherapy/brachytherapy [9], showing that the adoption of low-dose rate and high-dose rate interventional radiotherapy (HDR-IRT) techniques resulted in similar recurrence rate, although HDR-IRT was associated with a greater reduction in patients’ reported symptoms [10]. In addition, IRT improves the dose coverage to the target volume (TV), while reducing the dose to the surrounding Organs At Risk (OARs). As shown by Flickinger et al. [11], RT decreases the size of both normal and keloid scars, by acting on fibroblasts, mesenchymal cells and others rapidly growing inflammatory cells. Furthermore, the irradiation of the keloid scar in the post-operative setting acts on a more immature- and therefore radiosensitive- cellular and extracellular microenvironment as compared to the treatment of the mature tissue of the non-resected keloid [12].

However, available therapies have a low efficacy rate and the interest in studying risk factors is growing, leading to an increase in publication and citation rates on this topic [13].

Hence, the aim of our work is to investigate the role of post-operative interventional RT (POIRT) in a homogenous cohort of patients with keloids treated at a single institution, with the same fractionation schedule. The study will focus on reporting the results involving the combined surgical and POIRT approach, and on detailing the risk factors associated with recurrence.

## Patients and methods

### Data collection and inclusion criteria

The electronic institutional datasets were reviewed to identify patients who were clinically diagnosed with keloids and treated with surgical excision and POIRT following a clinical diagnosis of keloid from 2004 to 2020. Only patients with a histopathological diagnosis of keloid and a minimum follow-up of 24 months were included in the study. If a clinical assessment was not available, phone calls were used to evaluate local control and functional/aesthetic outcomes. The included patients had provided written informed consent for the use of anonymized clinical data for research purposes. This study was approved by our institutional and ethical commission (UID 3898).

Demographical, clinical and treatment data were collected for each included patient. The total active length of the POIRT was used as a surrogate measurement of the length of the surgical scar as the treatment was planned to cover the whole volume. Lesion location was categorized as breast, sternum, abdomen, ear, extremities, neck, thorax, and other (e.g. sacrum and gluteus). The presence of toxicity, recurrence during the follow-up, aesthetic outcomes, and status during the follow-up were also documented. Follow-up times were reported in months, and toxicities were classified as acute (occurring within 3 months from the end of HDR-IRT) or late (occurring after 3 months from the end of HDR-IRT), with erythema, itch, or pain being acute toxicities and fibrosis, dyschromia, hyperpigmentation, telangiectasia, diastasis, dehiscence, or other being late toxicities. Whenever available, the severity of toxicities was reported using the Radiation Therapy Oncology Group (RTOG) classification system [14].

### Surgery

Surgery was performed either in local or general anesthesia according to the length and width of keloid scar and/or number of keloids requiring treatment. Patients are admitted to the hospital for day surgery. The treatment began with a complete and proper surgical excision of the keloid tissue, avoiding the use of catgut sutures and electrocoagulation to minimize trauma to the wound bed and margins. All the removed scars underwent anatomopathological analysis. Hemostasis was carefully performed with bipolar forceps.

**Fig. 1:**
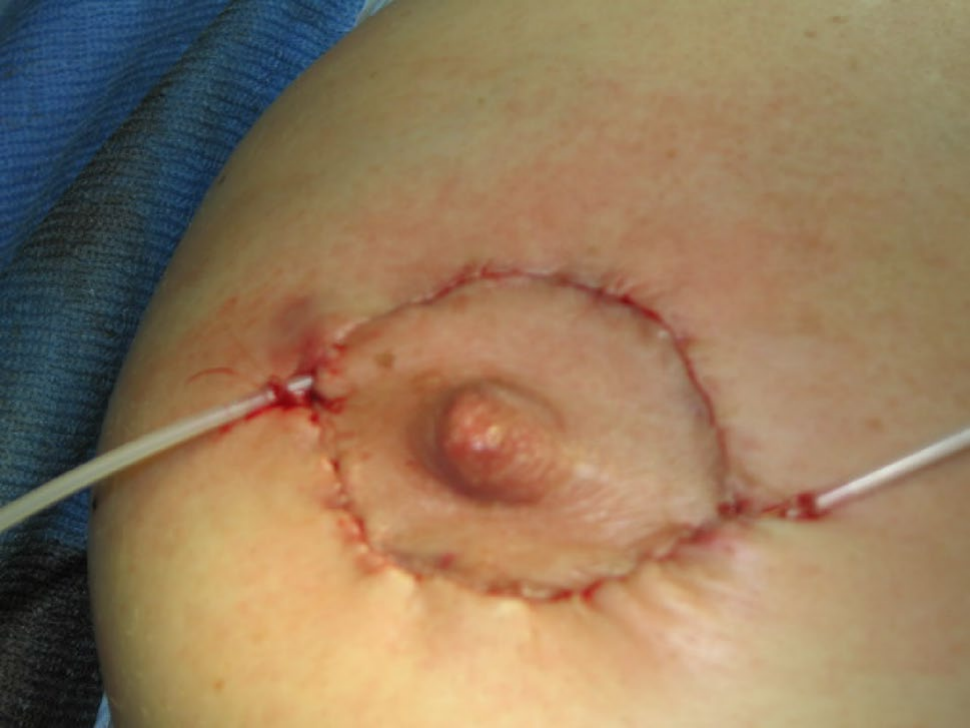
“In series catheters” positioning for a peri-areolar keloid

Direct suture of the wound margins was preferred to local flap reconstruction whenever possible. Local flaps were performed in case of large keloids causing large soft tissue defects, whose direct closure was not possible. The applicator-guide for brachytherapy (a standard flexible 5 or 6-french dedicated plastic tube) was inserted at 2–3 mm deep in the dermis, where the keloid originates, along the entire skin wound. At one end of the wound, the catheter was positioned subcutaneously in a closed pocket and exceeding the wound margin of approximately 5 mm. On the opposite side, the plastic tube was coming out from the wound itself. Example of positioning is available in Fig. 1. In addition to the interstitial technique, in a single case, a contact treatment with a personalized applicator (thermoplastic surface mould with embedded plastic catheters) was used to improve dose distribution.

### POIRT

After surgery, patients underwent computed tomography (CT) simulation with nonradioactive dummy-sources in the Radiation Oncology Department to confirm the correct position of the applicator. Brachytherapy dosimetry was performed using the Plato System (Elekta Nucletron) until December 2011 and the Oncentra Brachy (Elekta Nucletron) thereafter. The clinical target volume (CTV) encompassed the entire surgical wound. All the patients received a 3D treatment. The prescription dose was 12 Gy in 4 fractions (3 Gy/fr) administered twice a day with at least 6 h between fractions. A median distance of 5 mm from the source was chosen to minimize skin toxicity. Manual and/or graphical optimization was utilized to enhance the dose to CTV. POIRT was initiated within 4–6 h after surgery in all patients using a cable-driven Iridium-192 stepping source (micro-Selectron, Elekta Nucletron). After completion of treatment, the plastic catheter was removed. At the time of the treatment, the histopathological results of the specimen were not yet available due to the reduced interval between the surgery and POIRT.

### Statistical analysis

Continuous data were reported as median and ranges. Categorical data were reported as counts and percentages.

The cumulative incidence of recurrence curve function was estimated, and Ying and Wei variance estimator was used to account for within-patients correlation [15].

Univariable Cox proportional hazard regression models were used to evaluate the association of some patients’ and keloid scars’ characteristics and recurrence. A sandwich variance estimator was used to account for within-patients correlation.

All reported *p*-values were two sided, with *p*-value less than 0.05 considered as statistically significant.

All analyses were performed with the statistical software SAS 9.4 (SAS Institute, Cary, NC, USA) and R version 4.0.3.

## Results

Overall, a total number of 132 patients and 173 keloids treated between January 2004 and July 2020 were screened. Of these, 14 patients (21 keloids) were excluded due to the impossibility to retrieve a written informed consent, and 16 patients (17 keloids) could not be considered as the 24-month follow-up was not reached. Our study included seven patients who underwent two separate surgeries at different time points. Each surgery was treated as a distinct entity, resulting in a total of 109 surgeries being included in our analyses. The number of keloid scars operated in each surgery was as follows: 88 surgeries (80.7%) involved the treatment of a single keloid scar, 16 surgeries (14.7%) involved the treatment of two keloid scars, and 5 surgeries (4.6%) involved the treatment of three keloid scars. Finally, 102 patients and a total of 135 keloids were eligible for the analyses. Median age at the time of treatment was 43 years (range, 16–76), 83 (81.4%) patients were females and 19 (18.6%) males.

The leading cause of keloid formation was surgical treatment, accounting for 76.3% (100 cases) of the total, spontaneous formation accounted for 7.6% (10 cases), recurrence for 4.6% (6 cases), and other causes for 9.2% (12 cases). Additionally, re-irradiation was associated with 2.3% (3 cases) of keloid formation. The distribution of keloid scars across different anatomical sites was as follows: breast (33.3%, 45 cases), sternum (13.3%, 18 cases), abdomen (13.3%, 18 cases), ear (13.3%, 18 cases), limbs (6.7%, 9 cases), neck (5.2%, 7 cases), thorax (12.6%, 17 cases), and other sites (2.2%, 3 cases). The majority of cases involved the use of a single catheter (82.2%, 111 cases), while 16.3% (22 cases) had two catheters, and only 1.5% (2 cases) had three catheters. The median active length of the catheters used in the brachytherapy procedure was 7.0 cm, with a range from 0.8 to 32.0 cm.

A summary of surgical and clinical characteristics is provided in Tables 1 and 2 respectively.

**Table 1:** Descriptive variable at surgery level

Variable	Level	Overall (N = 109)
Number of keloid scars operated in each surgery, N (%)	1	88 (80.7)
	2	16 (14.7)
	3	5 (4.6)
Year of surgery, N (%)	2004–2007	22 (20.2)
	2008–2011	23 (21.1)
	2012–2015	39 (35.8)
	2016–2020	25 (22.9)
Age at surgery (y), median (min–max)		43 (16–76)

**Table 2:** Descriptive variable at keloid scar level

Variable	Level	Overall (N = 135)
Cause, N (%)	Surgery	100 (76.3)
	Spontaneous	10 (7.6)
	Recurrence	6 (4.6)
	Other	12 (9.2)
	Re-irradiation	3 (2.3)
	Missing	4
Site, N (%)	Breast	45 (33.3)
	Sternum	18 (13.3)
	Abdomen	18 (13.3)
	Ear	18 (13.3)
	Limbs	9 (6.7)
	Neck	7 (5.2)
	Thorax	17 (12.6)
	Other	3 (2.2)
Number of catheters, N (%)	1	111 (82.2)
	2	22 (16.3)
	3	2 (1.5)
Active length (cm), median (min–max)		7.0 (0.8–32.0)
Missing		2

Three patients with three keloids performed a second treatment with surgery and POIRT for a recurrence in the site of the first procedure (reirradiation subgroup). One patient, with an ear keloid, was treated using a hybrid HDR-IT and superficial-BT strategy due to the size and shape of the keloid and the very thin subcutaneous tissue in this region not allowing the insert of the classical plastic tube. Two women, who received the treatment for an abdomen keloid, became pregnant after POIRT and, despite this, they did not experience keloid recurrences.

No complication, such as bleeding or scar infections, occurred during treatment or after catheter removal.

The median follow-up was 64 [interquartile range, 25–103] months. In our cohort 36 (26.7%) recurrences were observed, distributed as follows: 2 (5.6%) were in neck, 2 (5.6%) in thorax (other than breast and sternum), 3 (8.3%) in breast, 11 (30.6%) in sternum, 2 (5.6%) in abdomen, 8 (22.2%) in ear and 8 (22.2%) in extremities.

**Fig. 2:**
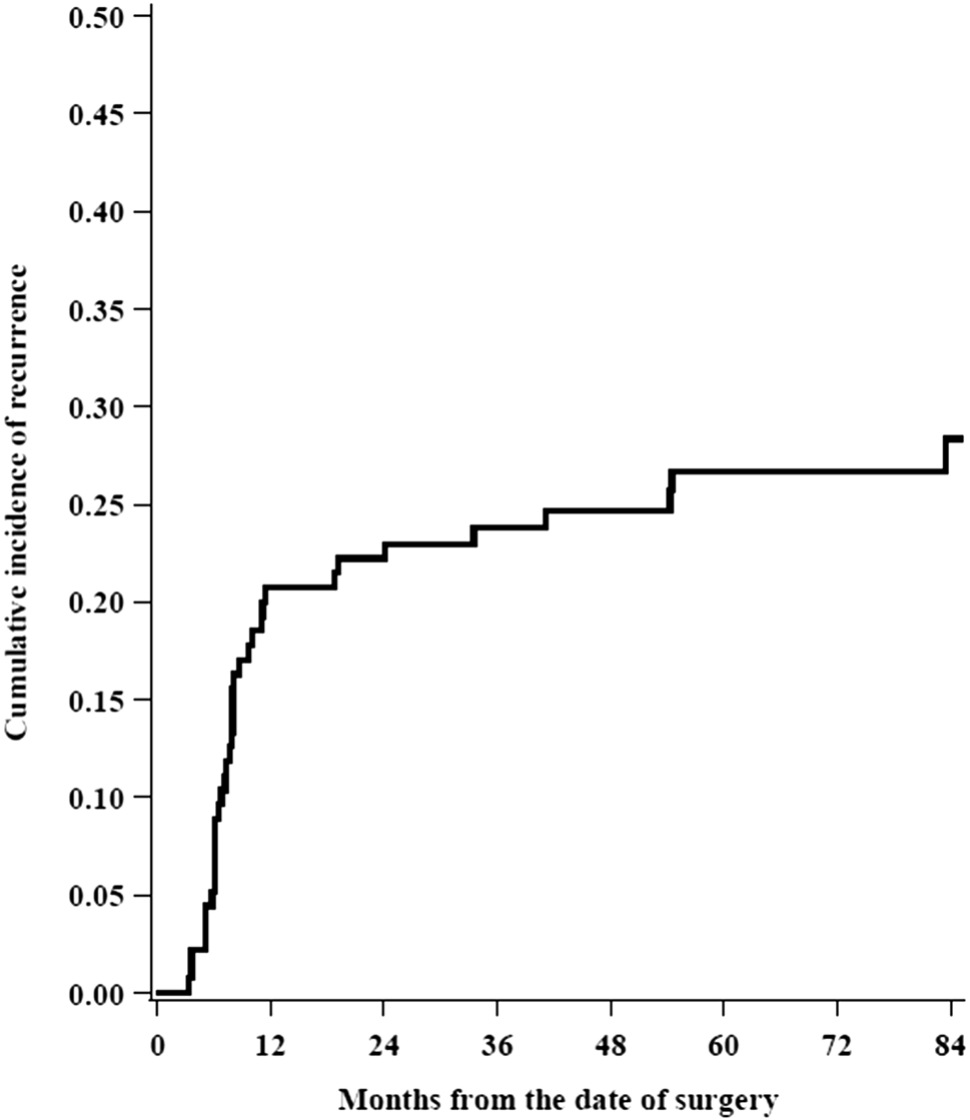
Cumulative incidence of recurrence

Twelve-months and 36-months cumulative incidence of recurrence were 20.7% (95% CI 12.2–28.5) and 23.8% (95% CI 14.9–31.7) respectively. Cumulative incidence of recurrence is showed in Fig. 2.

In the Univariate Cox Regression model, history of spontaneous keloids (HR = 7.00, 95% CI 2.79–17.6, *p* < 0.001), spontaneous keloid as keloid cause (HR = 6.97, 95% CI 2.05–23.7, *p* = 0.002) and sternal (HR = 10.6, 95% CI 3.08–36.8, *p* < 0.001), ear (HR = 6.03, 95% CI 1.71–21.3, *p* = 0.005) or limb (HR = 18.8, 95% CI 5.14–68.7, *p* < 0.001) keloid sites were significantly associated to a higher risk of recurrence. No significance has been show for sex (*p* = 0.10). The full variable analysis is available in Table 3.

**Table 3:** Univariable Cox proportional hazard regression models to evaluate the association of patients’ and keloid scars’ characteristics and recurrence

Variable	Level	N	Recurrence	HR	95% CI	P-value
Sex	Female	105	24	Ref	–	–
	Male	30	12	1.97	0.88–4.41	0.10
History of spontaneous keloids	No	121	26	Ref	–	–
	Yes	14	10	7.00	2.79–17.6	< 0.001
Cause	Surgery	100	20	Ref	–	–
	Spontaneous	10	7	6.97	2.05–23.7	0.002
	Recurrence/Re-irradiation	9	4	2.42	0.83–7.04	0.11
	Other	12	4	1.70	0.58–4.95	0.33
	Missing	4	1			
Site	Breast/Thorax	62	5	Ref	–	–
	Sternum	18	11	10.6	3.08–36.8	< 0.001
	Abdomen	18	2	1.42	0.23–8.71	0.70
	Ear	18	8	6.03	1.71–21.3	0.005
	Limbs	9	8	18.8	5.14–68.7	< 0.001
	Neck	7	2	4.22	0.42–42.3	0.22
	Other	3	0	N.e	N.e	0.77
Active length (cm)				0.98	0.92–1.05	0.59

Acute grade 1–2 erythema and itching were observed in 24 (17.8%) and 8 (5.9%) patients, respectively; 101 (74.8%) patients did not experience acute toxicity events. Chronic grade 1–2 toxicities were registered as follow: 15 (13.2%) fibrosis, 6 (5.3%) dyschromia, 4 (3.5%) diastasis and 1 (0.9%) telangiectasia. Six (5.3%) had both fibrosis and dyschromia and 8 (7.1%) had other toxicities; 74 (64.9%) patients didn’t experience chronic toxicity events. Missing data were reported for 21 patients. A positive aesthetic outcome, based on patients’ perception, was achieved in 51 (63%) out of 81 available cases. No grade 3 or greater toxicities were observed in both acute and chronic phases.

## Discussion

In recent years, several studies have explored the efficacy of postoperative radiation therapy with HDR-IRT in keloids scar treatment. Jiang et al. (2016) reported only 6% of recurrences after treating 32 keloids with 18 Gy in 3 fractions and a median follow-up of 29.4 months [16]. Bennet et al. (2017) [17] published a retrospective review to evaluate the role of surgical excision and PORT in 69 patients with 84 keloids. The overall RR was 27% and 74% for all keloids and for 31 keloids followed greater than 1 year, respectively. In the same year, Hanfkamp et al. published their research analyzing 24 patients with 29 keloids, mainly located in the ear, treated with a single 13 Gy-fraction POIRT; after a median follow up of 53 months, 24% of RR was reported [18]. Bijlard et al. (2018) conducted a retrospective multicenter comparison to determine the optimal fractionation for reducing keloid recurrences. They analyzed 238 keloids from three centers, with a maximum of 87 keloids per center. The fractionation and biologically effective dose (BED), calculated with an α/β ratio of 10 Gy, used in each center were: 9 Gy × 2 (34.2 Gy BED10), 6 Gy × 3 (28.8 Gy BED10), and 6 Gy × 2 (19.2 Gy BED10). The total recurrence rate for all keloids, including partial and full recurrences, was approximately 23%. Based on their findings, the authors concluded that the scheme using 6 Gy × 2 had similarly low recurrence rates and lower complication rates [19].

In 2022, Barragán et al. published their findings on 51 patients with 61 lesions who received HDR-IRT, reporting a recurrence rate of 4.9% when using 12 Gy in four fractions, with all recurrences being in the thorax. They suggested that increasing the dose at this location could be beneficial [20]. Liao et al. successfully treated seven complex keloids in three patients with HDR-IRT using 6 Gy × 3 fractions and observed no recurrences at a 9-month follow-up. They compared the dose distribution of HDR-IRT with that of treatment with electrons and found that HDR-IRT achieved higher coverage of the target than EBRT [21]. A systematic review and meta-analysis conducted by Zawadiuk et al. (2022) included 795 auricular keloids, revealing a recurrence rate of 9% after POIRT, 14% after excision with adjuvant compression therapy, 17% after excision with PORT EBRT, and 18% after excision with adjuvant steroid injections. The difference in recurrence rates among the four treatment groups was not statistically significant [22].

Our retrospective analysis of a large series of patients treated with POIRT, with a homogeneous fractionation showed a 26.7% rate of recurrence, which is slightly divergent from literature data. The reason for this difference may be related to the sample size of the patient population and the median keloids' size (expressed by active length) of about 8 cm. Indeed, in accordance with a recent review of Hsieh et al. [23], a length higher than 5 cm can be used as a cut off for a significant increased risk of relapse and the percentage of patients with a higher risk of relapse related to the spontaneous origin of keloids. It is worth noting that two patients who had undergone treatment for abdominal keloids subsequently became pregnant but did not experience any relapses. Although skin tension and hormone stimulation due to pregnancy are known to be important risk factors for keloid recurrence in this area [24, 25], there were no observed relapses in these cases.

The significance of having a minimum follow-up of 24 months was already established in 2001 by Guix et al. [26]. Both partial and full recurrences were classified as treatment failures in our series. Specifically, we observed partial relapse at the edges of the scar in certain anatomical sites, most likely due to suboptimal coverage of the surgical bed caused by dose leakage at the end of the catheters. Notably, in some patients, telephonic updates of follow-up information without any photos sent for direct evaluation by the physician could have led to an overdiagnosis of recurrences, even when only a hypertrophic scar was present.

The radiobiology of keloids is still a subject of debate. Some studies have hypothesized an α/β ratio of 10 Gy [27], while others have estimated it as low as 2 Gy [11], which could affect the response to different fractionations in the postoperative setting. The Biologically Effective Dose (BED) necessary for improved efficacy of radiation therapy is also debated. While some studies have shown that a recurrence rate below 10% is achieved when BED exceeds 30 Gy (α/β = 10 Gy), others have set the threshold at 20 Gy. Recent review suggests that a shorter interval between surgery and RT results in a lower recurrence rate [28], and a higher BED of 30 Gy is associated with better outcomes [27]. Based on preliminary results, we modified our fractionation to increase the BED, prescribing a dose of 5 Gy × 3 fractions, corresponding to a BED of 22.5 Gy and 52.5 Gy with α/β = 10 Gy and α/β = 2 Gy, respectively. In a de-escalation study, Renz et al. showed that the recurrence rate was lower in patients treated with a higher dose of 20 Gy in 5 fractions compared to those treated with inferior doses of 12 to 16 Gy in 3 to 4 fractions [29].

Notably, POIRT is just one of several strategies for the prevention of keloid recurrence [7], in fact, several studies have established the use of occlusive dressing, intralesional steroids, imiquimod and cryotherapy as effective alternatives [30]. Given these options, clinicians can tailor their approach to align with individual patient needs and the specific characteristics of the keloid, potentially even combining these techniques for optimal results.

This study has some limitations, including the wide range of time considered for patient inclusion, incomplete information on keloid size (active length used as surrogate measure), loss to follow-up, and potential overestimation of recurrences based on telephonic evaluation. However, the 16-year follow-up period with homogeneous fractionation is a unique strength, allowing for critical evaluation and potential improvements in efficacy.

## Conclusion

Our study evaluated the effectiveness of postoperative radiotherapy for keloid treatment using homogeneous fractionations. The findings support the use of surgery and POIRT as an effective strategy for controlling keloid relapses, as compared to other treatment options. Further research is needed to identify the optimal Biologically Effective Dose (BED) for keloid treatment and to establish a scoring system for patient selection for surgery and radiotherapy.
